# Unravelling *Anopheles* Dynamics in a Malaria-Free Paraguay: Species Distributions, Bioclimatic Niches, and Implications for Resurgence Risks

**DOI:** 10.3390/pathogens14090849

**Published:** 2025-08-26

**Authors:** Florencia del Puerto, Mauricio Grissetti, Luis Ferreira, Luciano Franco, Leidi Herrera

**Affiliations:** 1 Departamento de Biología Molecular y Biotecnología, Instituto de Investigaciones en Ciencias de la Salud (IICS-UNA), Universidad Nacional de Asunción, San Lorenzo 111421, Paraguay; fdelpuerto@iics.una.py; 2 Departamento de Biotecnología, Facultad de Ciencias Exactas y Naturales, Universidad Nacional de Asunción, San Lorenzo 111421, Paraguay; mauriciogrissetti@gmail.com; 3 Departamento de Entomología, Servicio Nacional de Erradicación del Paludismo (SENEPA), Ministerio de Salud Pública y Bienestar Social, Asunción 001221, Paraguay; luisferreirabio22@gmail.com (L.F.); lucianoentomologia@hotmail.com (L.F.); 4 Departamento de Medicina Tropical, Instituto de Investigaciones en Ciencias de la Salud (IICS-UNA), Universidad Nacional de Asunción, San Lorenzo 111421, Paraguay; 5 Instituto de Zoología y Ecología Tropical, Universidad Central de Venezuela, Caracas 1050, Venezuela

**Keywords:** *Anopheles*, malaria, DIVA-GIS, MaxEnt, Paraguay

## Abstract

Malaria, caused by a protozoan parasite of the genus *Plasmodium* and transmitted by mosquitoes of the genus *Anopheles*, remains a significant vector-borne disease worldwide. In 2018, Paraguay became the first country in the Americas in 45 years to be certified malaria-free by the World Health Organization. Between 2016 and 2017, a period with no reported human malaria cases, the presence of *Plasmodium* spp. in *Anopheles* mosquitoes was investigated in the departments of Caaguazú and Alto Paraná. These studies found that the most prevalent *Anopheles* species in Paraguay, including *Anopheles albitarsis* (59.4%), *Anopheles strodei* (21.5%), and other *Anopheles* species in smaller proportions, were all negative to the parasite. The objective of this study was to re-evaluate these presence data and to define environmentally suitable areas for *Anopheles* spp. and their association with bioclimatic variables using DIVA-GIS/MaxEnt software for the entomological surveillance of malaria risk in Paraguay. Results showed that areas of bioclimatic suitability included the Humid Chaco, Cerrado, Paraná Atlantic Forest, and Southern Cone Mesopotamian savanna ecoregions. The most relevant climatic variables were the precipitation of the wettest month (contribution of 80.4%) and the precipitation of the driest month (contribution of 18.4%). *Anopheles albitarsis*, also reported as a vector of the Venezuelan equine encephalitis virus in neighbouring countries, was the most abundant mosquito species. *Anopheles darlingi*, the main vector of malaria in Paraguay, was not found. However, species richness indices (Chao/ACE) suggest that cryptic or sibling species may be present. Finally, the possible succession of *Anopheles* species and their geographical segregation are discussed in scenarios of entomological surveillance and epidemiological risk.

## 1. Introduction

Malaria, caused by parasites of the genus *Plasmodium* (Apicomplexa: Plasmodiidae), is transmitted to humans by approximately 41 species of the genus *Anopheles* (Diptera, Culicidae, Anophelinae), with occasional zoonotic transmission from non-human primates [1].

According to the World Malaria Report [2], four countries in the Americas, Paraguay, Argentina, El Salvador, and Belize, have achieved malaria-free status since 2018. Paraguay’s experience exemplifies the success of coordinated public health interventions. Nonetheless, between 2012 and 2023, 72 imported cases were confirmed. The most prevalent species were *Plasmodium falciparum* (61%), *P. vivax* (33%), and *P. ovale* (6%). Notably, the first confirmed case of *P. ovale curtisi* in Paraguay was reported in 2013 in a traveller returning from Equatorial Guinea. Initially, molecular techniques (SnM-PCR and 18S rRNA sequencing) enabled the correct identification of the misdiagnosed *P. vivax*. The patient remained asymptomatic during follow-up, illustrating diagnostic challenges and the potential for latent infections [3,4].

The epidemiology of malaria transmission is tightly linked to the ecology and geographical distribution of its mosquito vectors, making entomological surveillance a cornerstone of control and elimination programs [5].

Since 1951, *Anopheles darlingi* has been reported in Alto Paraná and Alto Paraguay, later spreading to San Pedro, Villa del Rosario, and along the Paraguay and Paraná rivers. Its presence was subsequently confirmed in the Chaco region and near Lake Itaipú. Between 1955 and 1958, this species, typically found in southern Mexico and northern Argentina, established populations in seven Paraguayan departments: Caaguazú, Cordillera, San Pedro, Boquerón, Concepción, Alto Paraguay, and Presidente Hayes. By the 1980s, *An. darlingi* had become the primary malaria vector in Paraguay, significantly reshaping the country’s epidemiological landscape [6,7].

Recent findings confirming the presence of *An. darlingi, An. albitarsis*, and secondary vectors such as *An. strodei*, *An. triannulatus*, *An. argyritarsis*, and *An. fluminensis* across thirteen departments—including Central, Capital, Caaguazú, Canindeyú, and Presidente Hayes—highlight a broad current distribution and an ongoing risk of reestablishing local malaria transmission [4].

During the 1990s, intensified surveillance in high-risk areas reduced transmission, limiting endemicity to the eastern departments of Caaguazú, Canindeyú, and Alto Paraná. Nevertheless, persistent transmission near Yguazú Lake, an area with ecological conditions favourable for *Anopheles* breeding, remained a concern [8].

In 1999, however, Paraguay experienced a significant outbreak with 9943 *Plasmodium vivax* cases, though no fatalities occurred. Most eastern departments were then classified as medium- to high-risk zones. The epidemic outbreak prompted reinforced elimination strategies, and by 2005 the number of locally acquired cases had declined due to improved case detection, treatment, and surveillance of both local and imported infections [7,8].

On the other hand, although the Brazilian state of Acre does not directly border Paraguay, its high malaria endemicity (>1500 cases in 2025) poses an indirect threat via human mobility through the Amazonian regions of Bolivia and Peru. The existence of a transboundary geographic and human corridor connecting Argentina, Bolivia, and Brazil, and occasionally Paraguay, together with shared environmental conditions, increases the likelihood of imported malaria cases and vector mobility [4,9].

The continued presence of potential vector species and the effect of climatic change underscore the need for sustained entomological surveillance and assessment of bioclimatic suitability to prevent the reestablishment of malaria. Weather and climate are intrinsically linked to infectious diseases caused by protozoa, bacteria, and viruses [10,11,12].

Between 2016 and 2017, researchers from the Instituto de Investigaciones en Ciencias de la Salud (IICS) of the Universidad Nacional de Asunción (UNA) and the Servicio Nacional de Erradicación del Paludismo (SENEPA) surveyed historically endemic departments of Caaguazú, Alto Paraná, and Canindeyú to detect asymptomatic malaria and assess *Plasmodium* spp. circulation. While *Anopheles albitarsis* (59.4%), *An. strodei* (21.5%), and other species (19%) were identified, molecular diagnostics detected no *Plasmodium* infections in mosquitoes or humans [13]. Additionally, on the border with Brazil, PCR testing of non-human primate and bird samples—potential wild reservoirs—yielded no results [14].

The present study aims to re-evaluate the presence, abundance, and diversity of *Anopheles* spp. based on previous entomological data from tropical and subtropical humid forests within the Atlantic Forest biome. Using GIS modelling, associations with climatic variables were assessed to identify areas with current and potential suitability for vector presence in historically endemic and other at-risk regions [15].

Previous studies across South America [1,16] highlight the value of such approaches as tools for entomological monitoring and the assessment of potential vector succession with associated epidemiological risks.

## 2. Materials and Methods

### 2.1. Anopheles spp. Specimen Data

Records of the presence of different *Anopheles* species in the Caaguazú department (Santa Teresa, Coronel Toledo, Nueva Brasilia, Santa Clara, Mbarigui, San Juan Indígena, Nueva Esperanza, Pindo’í, and Mil Palos) and the Alto Paraná department (Nueva Esperanza, Misión Verbo Divino, and Mbaracayú) were collected in natural breeding areas using CDC and black Shannon traps between August 2016 and November 2017 (Paraguay’s warm spring season). These records were re-examined and georeferenced (see Supplementary Table S1). The database was cleaned of duplicate occurrences to remove sampling effort bias for distribution modelling. To reduce autocorrelation, only species separated by more than 1 km were selected.

### 2.2. Geographical Distribution Modelling

DIVA-GIS 7.3.0 (https://diva-gis.org/download.html, accessed on 20 February 2025) was used to model the distribution of *Anopheles* spp. by species, sampling area and department. Raster maps were produced showing the presence of *Anopheles* spp., areas of suitability for them, and bioclimatic niche matches. These maps used 19 bioclimatic variables (see Supplementary Table S2) from the Worldclim database (https://www.worldclim.org, accessed on 20 February 2025) with mean temperature, maximum and minimum temperature, and monthly rainfall interpolated for 50 years (1 pixel = 0.86 km^2^ in Ecuador as a reference point). An elevation layer with the same resolution was also considered.

The georeferenced *Anopheles* database was transformed into a csv file by converting the original grd format into a compatible ASCII format (*.asc) using MaxEnt 3.4.4 software (https://biodiversityinformatics.amnh.org/open_source/maxent/, accessed on 20 February 2025) [17] for maximum entropy inference of species niche distributions based on presence records. To mitigate overfitting problems and improve the ability to generalize to new data, and to provide a more accurate and realistic assessment of model performance, 75% of the data were reserved for training in MaxEnt. The model was run 10 times with 500 iterations. A total of 10,013 background points were used with 19 bioclimatic variables from Worldclim. DIVA-GIS 7.0 was used as the transformation platform to test whether the distribution of the different *Anopheles* species was random or could be predicted by a model such as MaxEnt. The results were analysed using the *Jackknife* test for the area under the curve (AUC) statistic to determine which bioclimatic variables influenced the predictive model [18]. The model’s performance was evaluated by successively removing each environmental variable and measuring the resulting change in model accuracy. The entire analysis was supported by Paraguay’s political divisions of hydrography and ecoregions (https://www.ine.gov.py/microdatos/cartografia-digital-2012.php, accessed on 20 February 2025).

The bioclimatic variables that were highly weighted in the models were compared with retrospective weather station information at the time of sampling, and analysed in terms of possible bio-ecological scenarios (https://www.ncdc.noaa.gov/cag/global/time-series, accessed on 29 July 2023). More specifically, temperature and precipitation rankings were available on a weekly timescale from 4 January 2020 to 29 July 2023.

### 2.3. Species Richness

The richness of *Anopheles* spp. within the study area was mapped using DIVA-GIS and represented by colour gradations; darker tones indicate higher species richness, while lighter tones indicate lower richness. “gis_osm-water_free-1” (from OpenStreetMap) provided open, collaborative data on bodies of water such as rivers, lakes, and streams.

Richness mapping was complemented by calculating the minimum number of species present at a 95% confidence level for the study area, including unobserved or underestimated species, using the Chao 1 95% lower bound method. The Abundance-Based Coverage Estimator (ACE), which is based on species abundance in records, was used to estimate richness by considering both rare and common species and focusing on how often they are observed. Both metrics were based on rarefaction curves, which assess the dynamics of species richness and how they change as more samples are collected [19].

### 2.4. Ethical Considerations

The study was part of the project approved by the Ethics and Scientific Committees of the Research Institute in Health Science of the National University of Asunción (IICS-UNA), Paraguay, under code M07/2024.

## 3. Results

### Anopheles spp.

The distribution of nine *Anopheles* species in the Caaguazú and Alto Paraná departments was mapped using DIVA-GIS based on a total of 916 records from 12 sampling sites between 2016 and 2017. Of these, 12% were georeferenced (see Supplementary Table S1).

**Figure 1:**
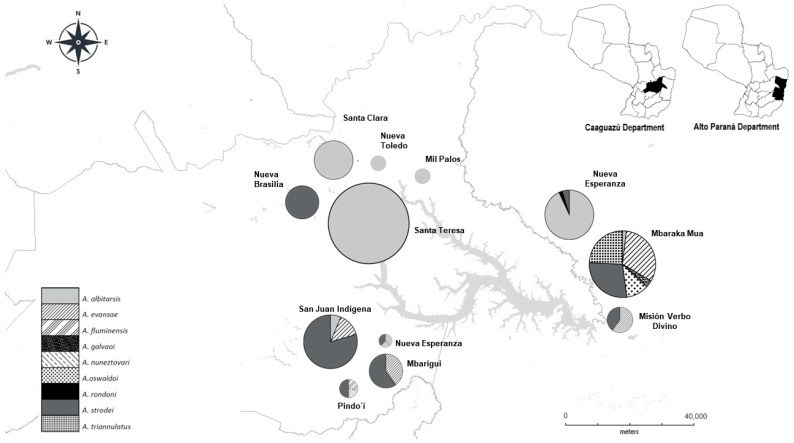
Map of distribution and abundance of *Anopheles* species in Caaguazú and Alto Paraná departments of Paraguay between 2016 and 2017 (DIVA-GIS). The abundance graphs show the proportion of each species collected (n): *An. albitarsis* = 554; *An. strode* = 200; *An. evansae* = 60; *An. oswaldoi* = 18; others = an average of six specimens. The circles or pizzas reveal the total percentage coverage, so each fraction for each species shows its percentage of the total by locality.

The most abundant species were *An. albitarsis* (*n* = 546), *An. strodei* (*n* = 200), and *An. evansae* (*n* = 88). The other species were present at an average of 14 specimens per species. Differences in distribution were observed in the north-west and south-west, using Lake Yguazú (Caaguazú) as a reference point, with a predominance of *An. albitarsis (n* = 424) and *An. strodei* (*n* = 142). In the central and south-eastern regions (Alto Paraná), two areas of the greatest species richness were identified, with Mbaraca Mua being the richest locality in all the samples. In Alto Paraná, *An. albitarsis* (*n* = 122), *An. evansae* (*n* = 61), *An. strodei* (*n* = 56), and other species such as *An. triannulatus, An. galvaoi*, and *An. oswaldoi* (*n* = 21 on average) were present (Figure 1).

**Figure 2:**
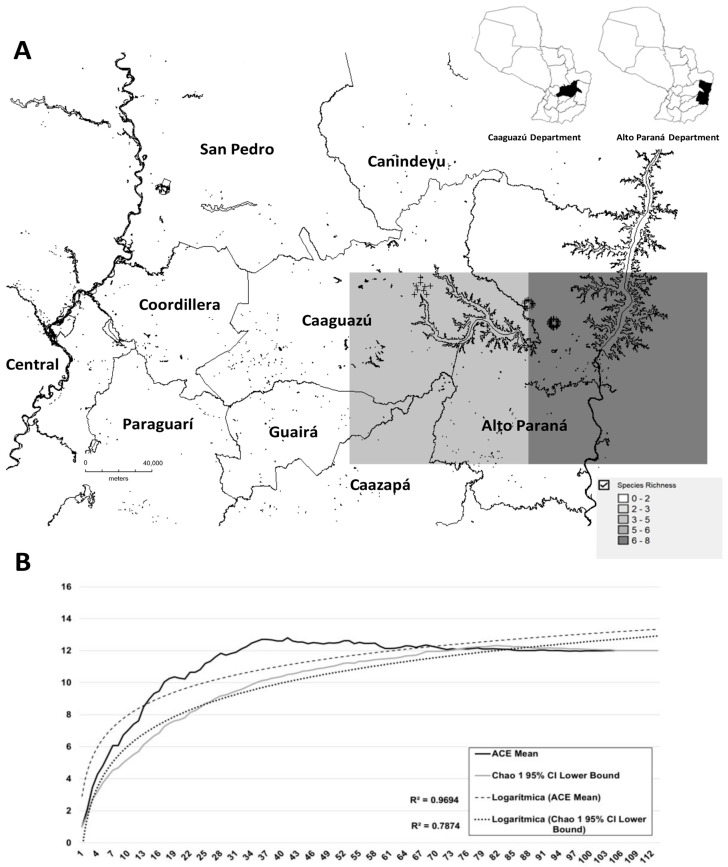
(**A**) DIVA-GIS representation of the real and potential richness of *Anopheles* species in Caaguazú and Alto Paraná, Paraguay, during 2016–2017. Quadrants ranging from white to intense grey reflect the number of species that can be found in areas of bioclimatic suitability. (**B**) Graph showing abundance estimators ACE and Chao 1 (*x*-axis = number of sampling events; *y*-axis = number of species collected).

The *Anopheles* species richness map, based on Chao 1 and generated using DIVA-GIS, showed regions of bioclimatic suitability for five to six species in Caaguazú, Guairá, Caazapá, and Alto Paraná (light grey grid image or presence cells, 178 × 114 km^2^), and six to eight species in Alto Paraná, a department close to the Paraná River and the border area (dark grey area, 101 × 112 km^2^, Figure 2A).

Rarefaction curves for Chao 1, based on presence/absence, showed an increase in species richness as more records were made. In 57% of the datasets, almost all species were already included, with values close to 65 and an R value of 0.7874, indicating a good fit.

The rarefaction curve for ACE exhibited a comparable trend, albeit with a higher R value (0.9694). Almost all species were registered at 37% of the records, with a plateau observed at 61%, all of which suggests a greater species richness estimate when rare species are included (Figure 2B).

**Figure 3:**
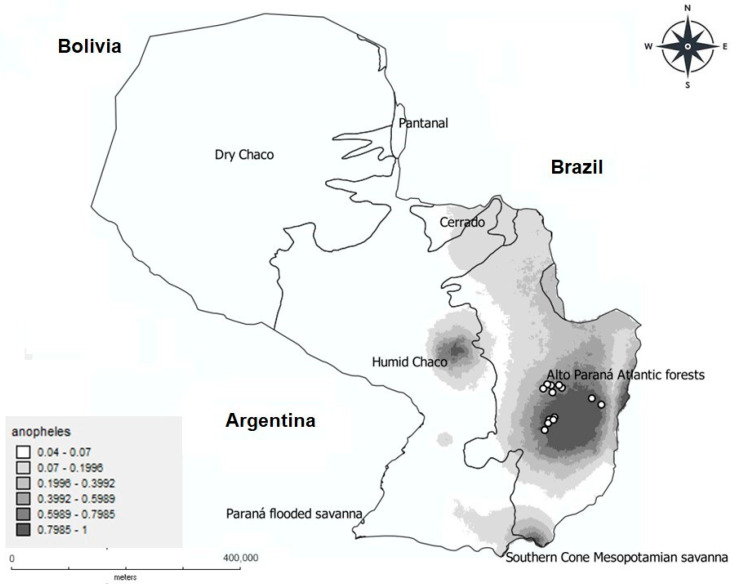
Distribution model of areas suitable for the real and potential presence of *Anopheles* species based on records in Caaguazú and Alto Paraná, Paraguay, using MaxEnt. The grey scale indicates the probability of species being present (from 0 to 1). White dots show the presence locations where the species were present and used for training.

MaxEnt modelling identified areas of bioclimatic suitability for the presence of *Anopheles*, including the Humid Chaco, the Cerrado, the Atlantic Forest in the north-east, and the Mesopotamian savannahs in the south. The absolute probability distribution of the data used in the model revealed an average value of 1.32 × 10^−3^ (*p* < 0.05). This suggests that the data distribution was not random but rather determined by bioclimatic variables and altitude. Figure 3 shows the probability of *Anopheles* spp. presence, irrespective of species, using a grayscale ranging from 0 to 1.

**Figure 4:**
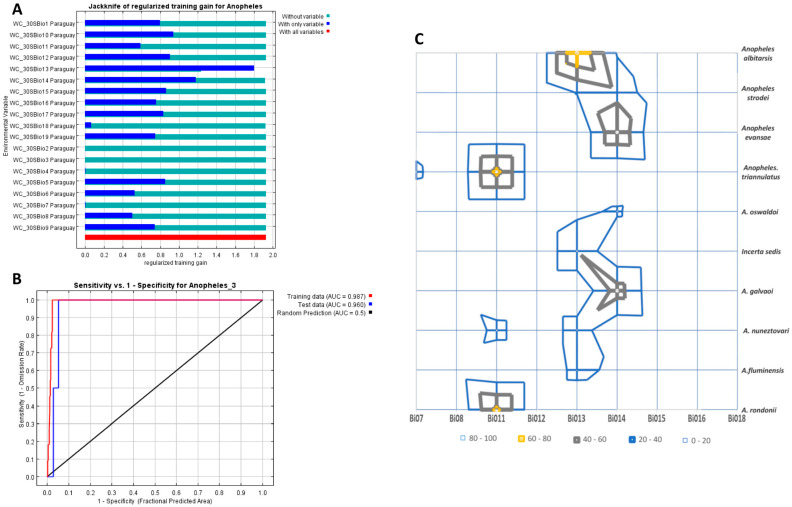
*Jackknife* test (AUC) for the MaxEnt model distribution of *Anopheles* species based on records in Caaguazú and Alto Paraná, Paraguay, as a function of Worldclim bioclimatic variables, altitude, and land use. (**A**). The *Jackknife* preponderant variables (13 and 14; abscissas) and their contribution to the distribution of species (ordinates). (**B**). Variation in sensitivity (ability of the model to identify areas suitable for the species to occur) versus specificity (ability of the model to identify areas where the species fails to occur). In the upper left corner, the asymptote is extended where sensitivity is 1 and specificity is 1. The high AUC (area under curve) gives high robustness. (**C**). Area diagram showing that Bio 13 and Bio 14 contributed between 60 and 80% to most species’ habitat suitability, except *An. triannulatus* and *An. rondoni*, which were determined by Bio b11 (mean temperature in coldest quarter).

After eliminating variables with low contribution and/or high correlation, the *Jackknife* test and the area under the curve (AUC) graph, in the MaxEnt model, revealed which bioclimatic variables were good predictors of conditions suitable for the presence of *Anopheles* species. The model fit was high (AUC-training data = 0.981; AUC-test data = 0.979). Figure 4A,B (as well as Supplementary Figures S1 and S2) illustrate these results.

The contribution of each selected bioclimatic variable to the estimation of the model was as follows: (i) precipitation of the wettest month (Bio 13, 188 mm per month at the time of the study) in 76.3%, (ii) precipitation of the driest month (Bio 14, 84 mm per month at the time of the study) in 17.9%, and (iii) annual precipitation (Bio 12, 115 mm per year at the time of the study) in 2.2%.

Similarly, a percentage graph showing the bioclimatic variables for the different areas of anopheline distribution, differentiated by species, revealed Bio 13 and Bio 14 were weighted between 60 and 80% for most species, except for *An. triannulatus* and *An. rondoni*, for which the Bio 11 variable (mean temperature of the coldest quarter) was dominant (Figure 4C).

## 4. Discussion

The presence of zoonotic disease vectors in countries with established geographical connectivity poses a significant epidemiological risk. The interconnected nature of the region facilitates the migration of vector species, which is often driven by human activity and commercial exchange. The fragility of health systems in these regions is a significant concern, as it increases their vulnerability [20].

Paraguay, once a malaria-endemic country in the 1940s, achieved a 99.9% reduction in locally acquired malaria cases by 2011–2012, fulfilling one of the Millennium Development Goals. This success reflects both sustained disease control efforts and the influence of specific bioclimatic conditions that allowed autochthonous transmission to persist in certain departments until 2011, despite its elimination elsewhere. However, the risk of imported malaria cases from Africa and Brazil via returning travellers remains a concern [21].

Imported malaria cases in Paraguay have steadily declined since 2012, with none reported in the past year. Most originated from Africa (mainly Equatorial Guinea), followed by Brazil and some Asian countries, affecting Paraguayan migrants. These cases occurred nationwide, especially in metropolitan areas, but did not overlap with areas of bioclimatic suitability identified in this study, suggesting minimal transmission risk pending the confirmation of low parasite load and vector density [6]. While current data suggest low transmission potential, entomological findings highlight the need for continued surveillance.

*Anopheles albitarsis* was spatially restricted to the north-central margin of Yguazú Lake, where few other mosquito species were recorded. In contrast, the south-eastern region showed a distinct ecological profile dominated by *An. strodei*, which co-occurs with at least ten other *Anopheles* species. Fluctuating water levels in the lake promote vegetation favourable to mosquito proliferation during critical thresholds, and have been linked to increased human malaria cases. A broader assessment of other water bodies—considering oxygen levels, solute concentrations, hydrological changes, and desiccation patterns in livestock areas—would enhance risk evaluation in Culicidae habitats. Similar approaches have been applied in other Southern Cone regions [16,22,23].

It is important to note that *A. albitarsis* has also been implicated in the transmission of Venezuelan equine encephalitis virus. This is an arbovirus that circulates in the Brazilian Pantanal (north-east of Paraguay) and the Argentine province of Corrientes (south-west of Paraguay), which underscores the necessity for vigilant surveillance of this species as, at the end of 2023, Argentina reported cases of Equine Encephalitis [22,24].

Although previous reports identified the study areas as ideal for *An. darlingi,* the main malaria vector in Paraguay and much of the Americas, this species was notably absent in the present study [22,25].

Abundance estimators identified almost all *Anopheles* species using only a third of the records, suggesting a consistent presence of species across departments. However, discrepancies between presence indices and abundance predictors suggest that broader sampling across more sites and time points could reveal cryptic species.

The region may be conducive to the emergence of sibling species such as *An. deaneorum* and *An. marajoara* [1,16]. Rarefaction curves showed that the ACE estimator required 37% of the records for representative sampling, whereas the Chao 1 estimator needed 57%. The higher ACE curve implies a greater estimated abundance of unsampled cryptic species, suggesting that more species may exist than were detected.

This study identified a suitable area for the *Anopheles* vector in central-eastern Paraguay, forming an ecological corridor across several ecoregions. These regions, home to significant Amerindian populations and relatively unspoilt environments, are vulnerable to urbanization and migration. Their historical association with malaria, coupled with recent case reports, highlights the importance of protecting these areas. This study sheds light on the dynamics of parasitic diseases in these endemic zones.

The present study’s modelling results align with other research findings regarding the potential distribution ranges of *Anopheles* species. Possible scenarios include the replacement of *An. darlingi* by other *albitarsis* complex species, as well as the emergence of cryptic species [1,8].

Species distribution modelling offers key insights into potential shifts in *Anopheles* dynamics and their roles as malaria vectors, aiding in assessments of disease resurgence in Paraguay. The bioclimatic variables used, mainly precipitation and temperature, are known to influence the mosquito life cycle, blood-feeding behaviour, and the *Plasmodium* sporogonic cycle, potentially raising transmission risk [23,26].

Despite being archived in surveillance institutions, information on malaria vectors in Paraguay remains scarce and is largely inaccessible to researchers [7]. This study significantly contributes to open data by using predictive software to generate information relevant to malaria surveillance and other *Anopheles* borne diseases.

Using MaxEnt and Diva-GIS modelling, areas suitable for vector development were identified. These areas align with regions that have historically experienced malaria endemicity, as documented by SENEPA, and could potentially extend to new areas. These findings improve our understanding of vector distribution and will support future risk assessments in Paraguay.

## Data Availability

Data are contained within the article.

## Supplementary Materials

The following supporting information can be downloaded at https://www.mdpi.com/article/10.3390/pathogens14090849/s1, Figure S1: Maxent modeling of *Anopheles* presence in Paraguay showing variable importance (Panel A), species response to key bioclimatic predictors, and model performance via ROC curve (Panel B); Figure S2: Pairwise correlations among bioclimatic variables (Bio1–Bio19) used in Maxent modeling of Anopheles distribution in Paraguay, highlighting significant associations (*p* < 0.05); Table S1: Georeferenced Records of Anopheles Species Collected in Paraguay by Locality and Department; Table S2: Description of Bioclimatic Variables (BIO1–BIO19) used in species distribution modelling.
