# Supplementary material for: Unravelling Anopheles Dynamics in a Malaria-Free Paraguay: Species Distributions, Bioclimatic Niches, and Implications for Resurgence Risks
Source: Pathogens. 2025 Aug 26;14(9):849. doi: 10.3390/pathogens14090849 (PMC12473100; doi:10.3390/pathogens14090849)
Supplement: Supplementary file 1 [file pathogens-14-00849-s001.zip › Table S2.pdf]

WorldClim

Home

- Historical climate data
- Historical monthly weather data
- Future climate data

Bioclimatic variables

Bioclimatic variables are derived from the monthly temperature and rainfall values in order to generate more biologically meaningful variables. These are often used in species distribution modeling and related ecological modeling techniques. The bioclimatic variables represent annual trends (e.g., mean annual temperature, annual precipitation) seasonality (e.g., annual range in temperature and precipitation) and extreme or limiting environmental factors (e.g., temperature of the coldest and warmest month, and precipitation of the wet and dry quarters). A quarter is a period of three months (1/4 of the year).

They are coded as follows:

BIO1 = Annual Mean Temperature

BIO2 = Mean Diurnal Range (Mean of monthly (max temp - min temp))

BIO3 = Isothermality (BIO2/BIO7) (×100)

BIO4 = Temperature Seasonality (standard deviation ×100)

BIO5 = Max Temperature of Warmest Month

BIO6 = Min Temperature of Coldest Month

BIO7 = Temperature Annual Range (BIO5-BIO6)

BIO8 = Mean Temperature of Wettest Quarter

BIO9 = Mean Temperature of Driest Quarter

BIO10 = Mean Temperature of Warmest Quarter

BIO11 = Mean Temperature of Coldest Quarter

BIO12 = Annual Precipitation

BIO13 = Precipitation of Wettest Month

BIO14 = Precipitation of Driest Month

BIO15 = Precipitation Seasonality (Coefficient of Variation)

BIO16 = Precipitation of Wettest Quarter

BIO17 = Precipitation of Driest Quarter

BIO18 = Precipitation of Warmest Quarter

BIO19 = Precipitation of Coldest Quarter
