# Supplementary figures and images for: Unravelling Anopheles Dynamics in a Malaria-Free Paraguay: Species Distributions, Bioclimatic Niches, and Implications for Resurgence Risks
Source: Pathogens. 2025 Aug 26;14(9):849. doi: 10.3390/pathogens14090849 (PMC12473100; doi:10.3390/pathogens14090849)

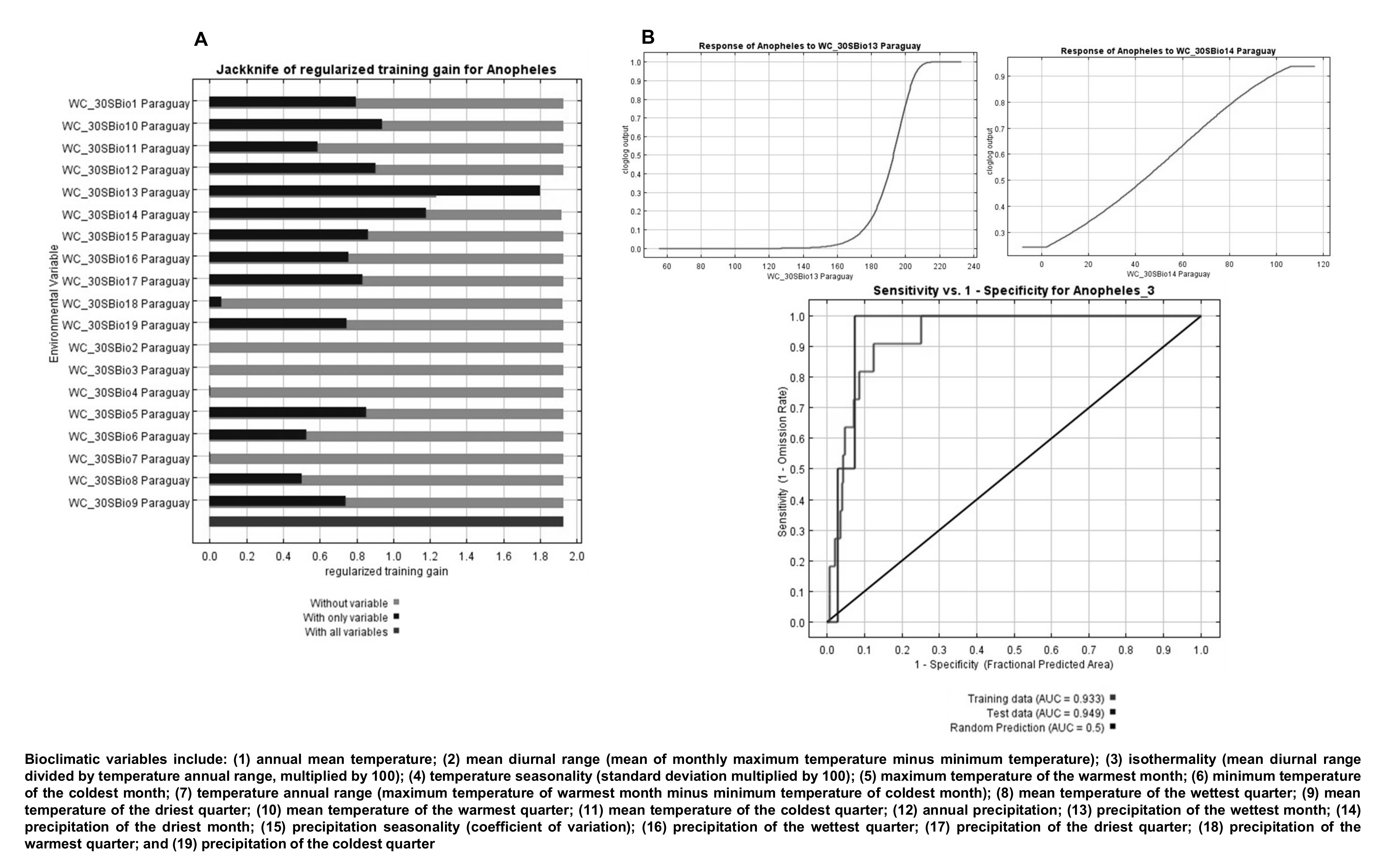

Supplement: Supplementary file 1 [file pathogens-14-00849-s001.zip › Figure S1.PNG]

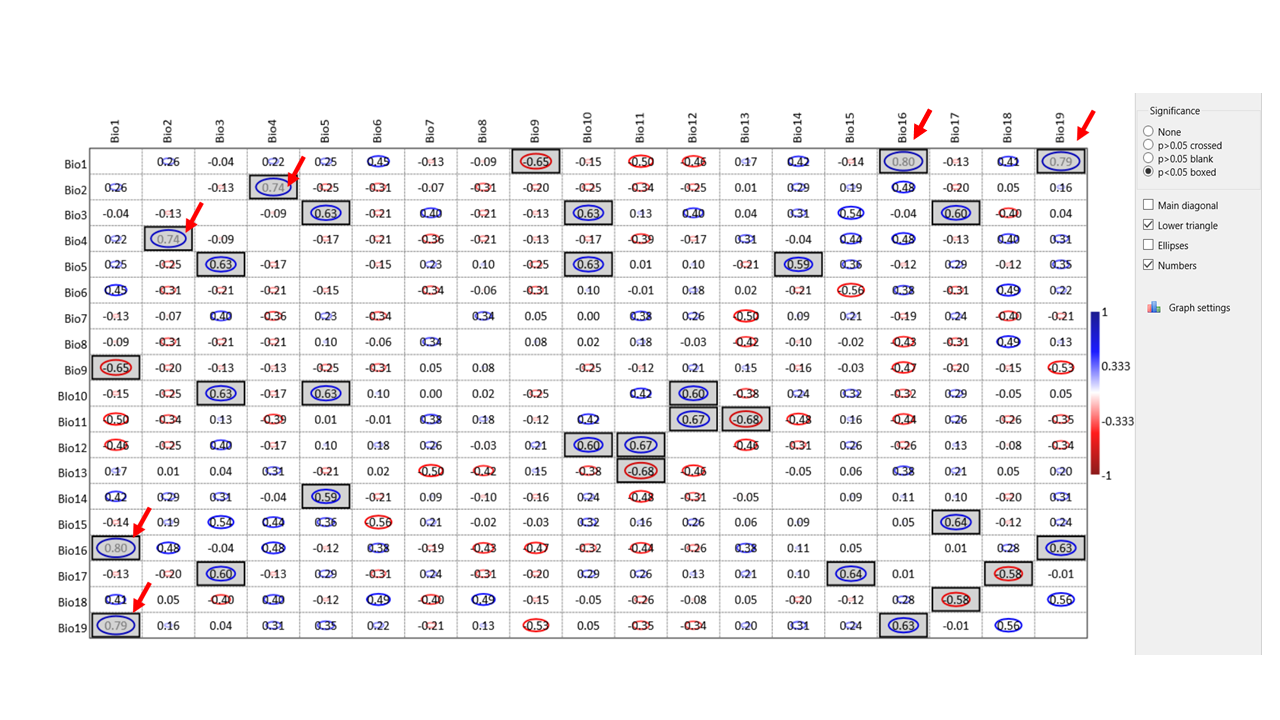

Supplement: Supplementary file 1 [file pathogens-14-00849-s001.zip › Figure S2.PNG]
